# P-1107. Oral Sulopenem/probenecid for Uncomplicated Urinary Tract Infections (uUTI): Results from the REASSURE Trial

**DOI:** 10.1093/ofid/ofae631.1295

**Published:** 2025-01-29

**Authors:** Sailaja Puttagunta, Steven I Aronin, Jayanti Gupta, Anita F Das, Kalpana Gupta, Michael W Dunne

**Affiliations:** Iterum Therapeutics, Old Saybrook, Connecticut; Iterum Therapeutics, Old Saybrook, Connecticut; Iterum Therapeutics, Old Saybrook, Connecticut; Das Consulting, Guerneville, CA; Boston University School of Medicine, Boston, Massachusetts; Iterum Therapeutics, Old Saybrook, Connecticut

## Abstract

**Background:**

Existing oral antibiotics for treatment of uUTI are not reliably effective due to rising antimicrobial resistant uropathogens. Sulopenem is a broad-spectrum IV/oral penem being developed for treatment of multidrug resistant infections. We conducted a pivotal Phase 3 randomized, double-blind, double-dummy, active controlled trial to evaluate the safety and efficacy of sulopenem/probenecid (SUL) vs amoxicillin/clavulanate (AMC) for treatment of uUTI.

**Figure:**
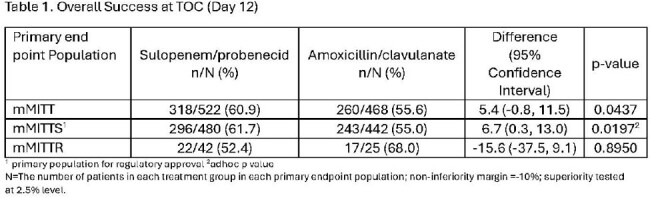

**Methods:**

Adult women with uUTI were randomized to SUL or AMC, both bid for 5 days. The primary objective was to establish noninferiority of SUL to AMC in the mMITT population (patients with ≥10^5^ CFU/mL Enterobacterales in baseline urine culture). The primary endpoint was overall success (combined clinical and microbiologic success) at the Test of Cure (TOC) visit. Using a pre-specified procedure to control for multiplicity, mMITT patients with baseline pathogens susceptible to AMC (mMITTS) were tested for noninferiority/superiority and those with nonsusceptible pathogens (mMITTR) were tested for superiority.

**Results:**

Of 2222 women randomized, 990 (44.6%) had ≥10^5^ CFU/mL Enterobacterales in baseline urine cultures and were in the mMITT population (Table 1). Of these, 922 had pathogens susceptible to AMC (mMITTS). The sample size in the mMITTR population (N=67) was lower than anticipated and not sufficiently powered to draw any conclusions.

Treatment emergent adverse events (TEAE) occurred more frequently in SUL treated patients (all, 18.9% vs 12.3%; related, 14.0% vs 7.7%) with the most frequent TEAEs being diarrhea, nausea and headache. Premature discontinuation from study drug due to TEAEs was low in both treatment groups (≤1%). No serious adverse events were reported in the SUL group, while 5 (0.5%) occurred in the AMC group.

**Conclusion:**

Sulopenem/probenecid was non-inferior to AMC for the treatment of adult women with uUTI in the mMITT population. In the mMITTS population, SUL demonstrated non-inferiority and based on an ad hoc analysis, demonstrated superiority to AMC. SUL was well tolerated with a safety profile consistent with other β-lactams and has the potential to fill the substantial unmet medical need for an empiric oral antibiotic option for outpatients with uUTI in this era of rising antimicrobial resistance.

**Disclosures:**

**Sailaja Puttagunta, MD**, Iterum Therapeutics: Employee|Iterum Therapeutics: Employee|Iterum Therapeutics: Stocks/Bonds (Public Company)|Iterum Therapeutics: Stocks/Bonds (Public Company) **Steven I. Aronin, MD**, Iterum Therapeutics: Employee|Iterum Therapeutics: Employee|Iterum Therapeutics: Stocks/Bonds (Public Company)|Iterum Therapeutics: Stocks/Bonds (Public Company) **Jayanti Gupta, PhD**, Iterum Therapeutics: Advisor/Consultant **Anita F. Das, PhD**, Cidara: Advisor/Consultant|Contrafect: Advisor/Consultant|Iterum Therapeutics: Advisor/Consultant|Paratek: Advisor/Consultant|Utility therapeutics: Advisor/Consultant **Kalpana Gupta, MD**, Iterum Therapeutics: Advisor/Consultant **Michael W. Dunne, MD**, Iterum Therapeutics: Advisor/Consultant|Iterum Therapeutics: Board Member|Iterum Therapeutics: Stocks/Bonds (Public Company)

